# Supplementary material for: From sequence to enzyme mechanism using multi-label machine learning
Source: BMC Bioinformatics. 2014 May 19;15:150. doi: 10.1186/1471-2105-15-150 (PMC4229970; doi:10.1186/1471-2105-15-150)
Supplement: Additional file 2 — Java code of ml2db. Additional file ml2db_code.tar.gz contains the Java source code to run the multi-label machine learning experiments and save the results to database. The code’s Javadoc is included. [file 1471-2105-15-150-S2.zip › additional file 2/ml2db/ml2db/doc/main/ml2db/al/utility/neighbours/package-summary.html]

main.ml2db.al.utility.neighbours


JavaScript is disabled on your browser.


- Overview
- Package
- Class
- Use
- Tree
- Deprecated
- Index
- Help

- Prev Package
- Next Package

- Frames
- No Frames

- All Classes

# Package main.ml2db.al.utility.neighbours

- Class Summary

  | Class | Description |
  | --- | --- |
  |  |  |
  | --- | --- |
  | LinearMultiLabelNNSearch | Class |
  | LinearNNSearchSkipInstanceItself | Class |
  | Neighbourhood | Contains information about: an instance, how much it is needed in this data set (the instances it is the nearest neighbour of), and how much it is labelled (whether it or its nearest or further neighbours are already labelled or partially known). |
  | NeighbourhoodExperimentDetails | Class |
  | NeighbourhoodExperimentDetailsSerialised | Class that uses an already calculated neighbourhood network to run an active learning experiment. |
  | NeighbourhoodNetwork | A collection of instances "needed" to label other instances. |

- Overview
- Package
- Class
- Use
- Tree
- Deprecated
- Index
- Help

- Prev Package
- Next Package

- Frames
- No Frames

- All Classes
